# Change of Mechanical Properties of Powder Recyclate Reinforced Polyolefin Based on Gamma Radiation

**DOI:** 10.3390/polym9090384

**Published:** 2017-08-23

**Authors:** Yilmaz Kismet

**Affiliations:** Department of Mechanical Engineering, Munzur University, 62000 Tunceli, Turkey; ykismet@munzur.edu.tr; Tel.: +90-428-213-1794 (ext. 2410)

**Keywords:** thermoplastic, polyethylene, polypropylene, electrostatic powder coating waste, gamma radiation, mechanical properties

## Abstract

In this study, the changes observed in the mechanical properties of standard test specimens that were produced with powder coating reinforced polyolefin (polyethylene and polypropylene) due to gamma radiation were examined. Matrix material of these specimens included low density polyethylene and polypropylene and 5%, 10%, 20%, 30%, 40% and 50% electrostatic powder coating waste by weight, and the samples were exposed to 44 kGry gamma-radiation for twenty four hours. Mechanical tests applied to the specimens after radiation demonstrated that the physical bonding mechanism between matrix material and filler material was reinforced. In the mechanical tests, tensile strength, three-point bending strength, and Izod impact strength of the samples were investigated and the results were compared to the results obtained in the mechanical tests when they were not radiated. Thus, the effects of gamma radiation on the mechanical properties of the filler material, and the electrostatic powder coating reinforced polyethylene and polypropylene were determined. Furthermore, section images of the radiated samples were taken with a scanning electron microscope and compared to the section images of irradiated samples to observe the physical bonding mechanism.

## 1. Introduction

Human needs grow along with the continuously increasing world population and the technological advances. To be able to respond to these demands, today, it has become significant to utilize existing waste, alternative materials, and various recycling techniques along with the technological advances. The most important alternative materials are the polymer-based materials that we have been using for several years in all areas of our lives. Polymers and polymer-containing composites are currently preferred in several industrial fields, and products that are easy to produce, low in cost, and able to meet the consumer demands could be developed with these materials [1,2,3].

Polymers are used in the pure form in industries, in addition to the several polymer matrix composites that have been developed through reinforcement with various organic and inorganic additives when needed [1,2,3,4]. Thus, it is possible to improve any negative property of a product obtained from polymers, and at the same time the production cost could be reduced by facilitating the production. For instance, organic materials such as hemp, flax, and jute are used in industry to improve the thermal, mechanical or other physical properties of several polymer based products [5,6,7,8]. As an example, furniture parts are produced by mixing sawdust with polymer-reinforced adhesives and pressing [9]. In mixes, the main composite ingredients are called matrix material, while the other elements used to improve any desired property of the matrix material are called reinforcement or filler materials [5,6,7,8,9,10,11,12]. To obtain an adequately strong physical bond between the matrix and filler mixtures the obtained mixture should be as homogenous as possible. This physical interaction and homogeneity has a direct impact on many different properties of the produced composite, primarily on its mechanical, thermal, and physical properties [5,6,10,13,14,15,16,17]. Bonding mechanisms are reinforced with different binding elements (Genomer, Elastolan, maleic anhydride) in poor binding composites [18,19,20].

The most important group of polymers includes thermoplastics. Thermoplastics are solid at ambient temperature and become soft and viscous when heated. It is possible to heat and cool thermoplastics several times without causing any deformation. An important group of thermoplastics includes polyolefins, and like other thermoplastics, they could be softened with heat and thus flow and then after forming under pressure and cooling, the new material would take the original solid and hard form of the polymer. As examples of the most preferred polyolefin types in the market, polypropylene (PP), polyethylene (PE), polymethylpentene (PMP) and polybutene-1 (PB-1) could be given [1]. In the present study, polyethylene and polypropylene matrix material, the main members of the polyolefin group, were used in standard test specimens [1,2,3,4].

Electrostatic powder coatings are frequently used to coat metal and non-metallic surfaces. These are synthetic dyes and do not contain any chemical solvents [21,22]. In practice, the surfaces to be coated by the electrostatic method are sprayed with a powder coating spray gun. Powder particles that are loaded in the gun attach to the surface to be coated due to electrostatic attraction. It is possible to re-use the unattached powder particles that pass through in low efficiency mass production systems [21,22,23,24,25,26]. Furthermore, thermoset epoxy/polyester systems are used as thermoplastic filler material after hydrolysis. Thus, changes in the mechanical properties of the samples produced with powder-reinforced polyethylene and polypropylene could be determined [13]. In the present study, the objective was to determine the changes in the mechanical properties of standard tensile test specimens produced with electrostatic powder coating waste and polyethylene and polypropylene mixtures with gamma radiation and the test results to be compared to the mechanical properties that were present before exposure to gamma radiation. Thus, the effects of the gamma radiation on the mechanical properties of the specimens obtained with matrix and filler material mixtures would be determined.

Electromagnetic radiation, gamma radiation is an application used in the polymer industry, albeit not actively. Gamma radiation is preferred mostly for the sterilization of plastic medical products, and this process is conducted at low doses without causing any molecular disruption. Furthermore, gamma radiation is used in applications to reinforce the bonding mechanisms in polymer composites [20,27]. For the reinforcement of polymer composites performed at higher doses when compared to sterilization applications, an average of 30 to 150 kGy gamma radiation is applied [1,2,27]. Thus, the intermolecular bonds in the matrix material that form the composite strengthen prior to degradation. Due to their molecular structure, polyethylene and polypropylene have a radical network structure and there is a need for a gamma radiation of about 50 to 100 kGy to reinforce their network structures by stimulating these mechanisms. Different physical properties of the polyethylene and polypropylene composites could be improved to a desired level with the effect of the applied gamma radiation on the polymer material since they absorb the radiation completely without reflecting it [20,27].

In the present study, the effect of gamma radiation on the mechanical properties of powder recyclate reinforced polypropylene and polyethylene was investigated. Radiation findings are displayed in graphs and discussed by comparison with the pre-radiation mechanical findings.

## 2. Materials

**Figure 1:**
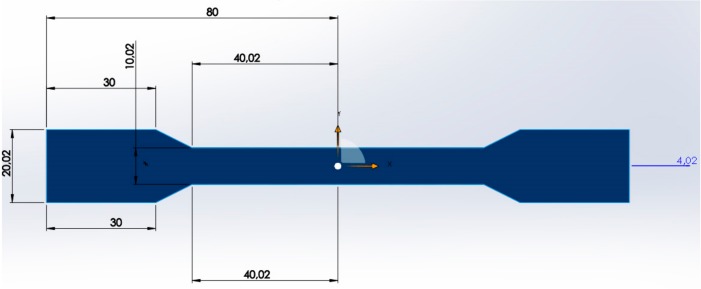
Standard test specimen.

The matrix material used in the standard test specimens given in Figure 1 was low density linear polyethylene (LLDPE) produced by “Exxonmobile” corporation. Another utilized matrix material was polypropylene (PP), which was produced by Sabic corporation with the product code 579S [13]. The hydrolyzed polyester-epoxy system electrostatic powder coating waste was used as the filler material in standard tensile specimens. Furthermore, 5% maleic anhydride (MAH) by weight produced by “Exxonmobile” with the product code “Exxolar PO 1020” was used as a binder in the production of standard tensile specimens that contained 40% and 50% filler by weight and were exposed to the gamma radiation [13]. Since the utilized MAH was grafted PP, in other words, it was polypropylene based, it was used only in PP-matrix mixtures.

Polyethylene matrix specimens produced in the plastic injection machine including 5%, 10%, 20%, 30% filler by weight and polypropylene matrix standard tensile test specimens also containing 10%, 20%, 30%, 40% and 50% filler and polyethylene and polypropylene test specimens that did not contain any filler material were classified as indicated in Table 1 and Table 2. Then, the classified standard test specimens (180 in total) were exposed to gamma radiation at the Turkish Atomic Energy Agency (TAEK) in Ankara. The 44 kGy gamma radiation was applied to the samples for 24 h.

Mechanical analysis of the classified specimens that were also exposed to gamma radiation was conducted without changing the classification. Mechanical analyses included tensile stress, three point bending strength and Izod impact strength tests conducted on the specimens. In the mechanical analyses performed for each mixing ratio, a total of 15 specimens were used (for each analysis 5 specimens) as indicated in Table 1 and Table 2. The results obtained in the above mentioned tests and the results of the tests conducted on the specimens that were not exposed to gamma radiation were tabulated and compared.

**Figure 2:**
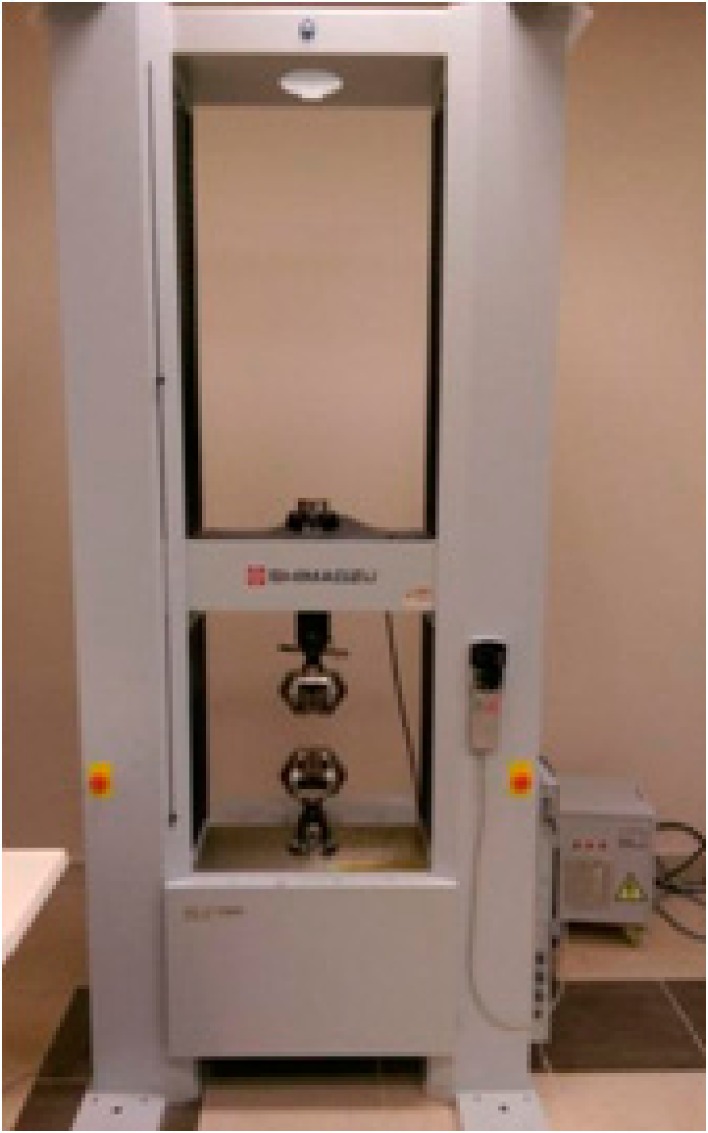
Shimadzu ag-x 10-ton Draw Bench.

The tensile strength of the specimens was tested in accordance with DIN EN ISO 527 standard at a speed of 50 mm/min under the impact of a 2 N front force. Three-point bending strength was tested in accordance with DIN EN ISO 178 standard with a 10 mm speed per minute and a maximum bending of 6 mm. In both analyses, the Shimadzu ag-x 10-ton machine shown in Figure 2 located in Munzur University Mechanical Engineering laboratory was used [1,2].

The Izod impact strength tests were conducted using a “Veb” brand impact device under ISO 180 standards, and the results are given in KJ in related graphs.

Cross sections of certain specimens were imaged using an electron microscope to better observe the physical bonding mechanism between the matrix and filler. This procedure was conducted using the ‘jeol jSM 7000f field emission’ device located at Fırat University Physics Department/Material Science laboratory. The materials were dehumidified in the incubator for 24 h before examination in the electron microscope and the surfaces were coated with gold.

## 3. Results and Discussion

### 3.1. Mechanical Properties

The results demonstrating the effects of gamma radiation on the mechanical properties of filler-reinforced polyethylene and polypropylene are given in the following graphs. Furthermore, the comparison of the abovementioned results and the results obtained with specimens not exposed to gamma radiation are tabulated and interpreted.

### 3.2. Tensile Test

#### 3.2.1. Tensile Stress Results for Polyethylene

**Figure 3:**
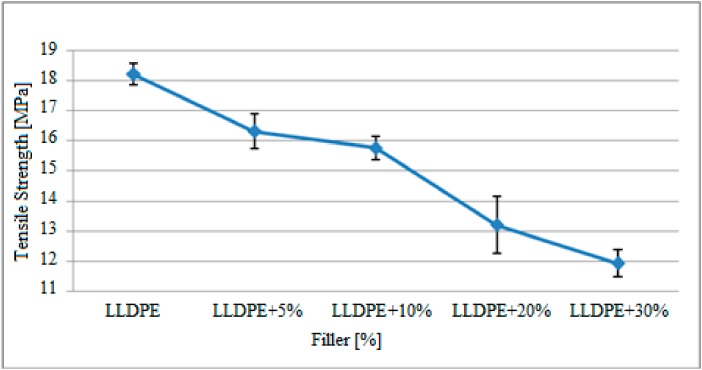
The variations in tensile stress of the polyethylene matrix specimens after gamma radiation based on the filler material. LLDPE = low density linear polyethylene.

The change observed in the tensile stress of the polyethylene matrix specimens with the mixing ratios shown in Table 1 after gamma radiation is presented in Figure 3.

Based on the graph above, the tensile stress of the polyethylene specimens exposed to gamma radiation decreased based on the increase in filler material. The tensile strength of the pure polyethylene specimens without filler was approximately 18.2 MPa, while this figure decreased with increasing filler material content and was calculated as 11.9 MPa for specimens containing 30% filler. It was observed in the graph that the maximum standard deviation range occurred in 20% filler reinforced specimens. The large standard deviation range could have occurred due to fact that the matrix and filler material might not have mixed homogeneously and air voids could have formed in the produced specimens.

These results are presented in Table 3 to compare with the tensile stress results of the study by Kısmet (2012) conducted with specimens that were not subjected to gamma radiation.

Based on the findings presented in Table 3, the tensile stress of the specimens improved after the gamma radiation. The tensile stress of the pure polyethylene specimen without filler increased from 17 MPa to 18.2 MPa after gamma radiation. Similarly, tensile strength of the specimens with 30% filler material increased from 10.9 MPa to 11.9 MPa, a 1.0 MPa increase. That is, the tensile strength of the gamma radiated samples improved about 10–20% when compared to the pre-radiation state. Also, in general, the difference in the tensile stress of pure polyethylene specimens and specimens with 30% filler remained almost the same before and after radiation. The change in tensile stress for specimens not exposed to gamma radiation was 6.1 MPa and it was 6.3 MPa for gamma radiated specimens.

#### 3.2.2. Tensile Stress Results for Polypropylene

**Figure 4:**
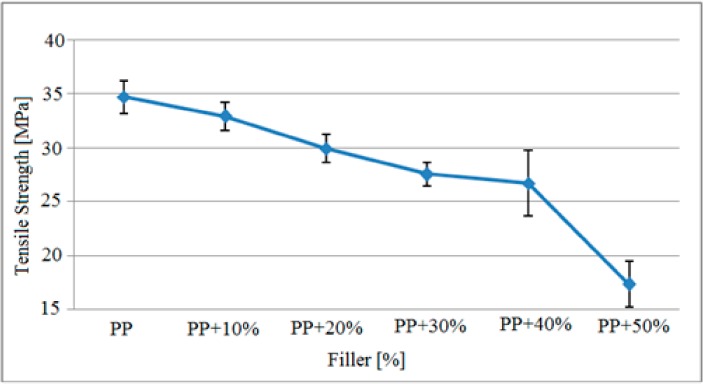
The variations in tensile stress of the polypropylene matrix specimens after gamma radiation based on the filler material.

The variation in the tensile stress of the filler-reinforced polypropylene matrix specimens at different rates and exposed to gamma radiation shown in Table 2 is presented in Figure 4.

As indicated in Figure 4, the tensile stress of the polypropylene matrix samples exposed to gamma radiation decreased based on the increase in filler material. The tensile strength of pure polypropylene specimens was approximately 34.7 MPa, which was reduced to 27.5 MPa for samples containing 30% filler and 17.3 MPa for samples containing 50% filler material based on the increase in filler material. Also, it could be observed that the samples with 40% and 50% filler did not have sufficient homogeneity in their production and there were a high number of air voids in the specimens, as reflected by the high standard deviation range.

**Figure 5:**
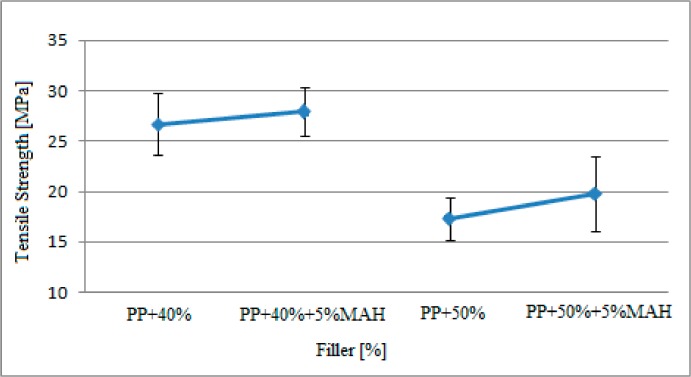
The variation in tensile stress of polypropylene matrix samples based on filler and maleic anhydride (MAH) content after gamma radiation exposure.

The tensile strength findings on the specimens “G” and “H” containing 5% MAH (maleic anhydride) and exposed to gamma radiation show in Table 2 are presented in Figure 5.

Based on the findings given in Figure 5, the tensile stress of the specimens, with the same filler ratio and exposed to gamma radiation, improved with the addition of MAH. The tensile stress of the 50% filler-reinforced polypropylene matrix specimens was approximately 17.3 MPa, while the tensile stress of the polypropylene matrix samples containing the same rate of filler and 5% MAA was approximately 19.8 MPa.

The findings observed in Figure 4 and Figure 5 are presented in Table 4 compared with the tensile stress results of the study by Kısmet (2012) conducted with specimens that were not subjected to gamma radiation.

Based on these findings presented comparatively in Table 4, it was observed that the tensile stress of the specimens was improved with radiation exposure. The tensile stress of pure polypropylene without filler increased from 32.8 MPa to 34.7 MPa. Similarly, the tensile stress of polypropylene samples containing 50% filler material increased from 3.4 MP to 14.3 MPa based on the gamma radiation. Furthermore, the tensile stress of the specimens containing 50% filler material and 5% MAH and not exposed to gamma radiation increased 3.9 MPa after radiation; from 15.9 MPa to 19.8 MPa.

### 3.3. Bending Strength

#### Polyethylene Bending Strength Findings

**Figure 6:**
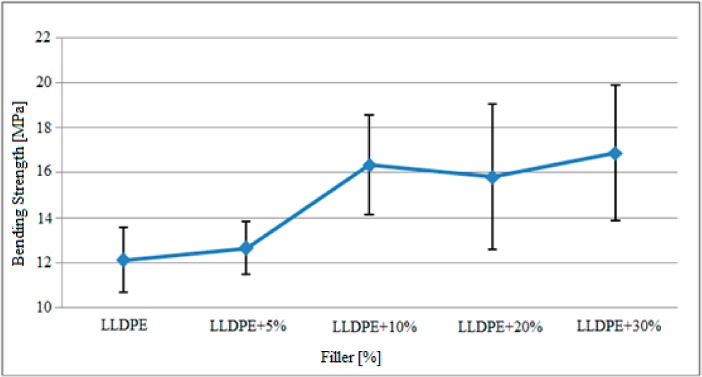
Variation in bending strength of polyethylene matrix specimens after gamma radiation based on the filler material.

The variation in three point bending strength of the polyethylene matrix samples with the mixing ratios depicted in Table 1 after gamma radiation is presented in Figure 6.

Based on the graph above, the bending strength of the specimens after radiation increased with the increase in filler content contrary to the tensile stress. Based on these findings, the bending stress of the polyethylene samples without filler was about 12.1 MPa on average and increased with the increase in filler content and reached approximately 16.9 MPa in 30% reinforced samples.

These results are compared with the three point bending strength findings for the non-gamma-radiated polyethylene specimens in Table 5.

Based on the findings given in Table 5, it was observed that the strength of the specimens against the vertical loads increased with the gamma radiation exposure. The bending strength of pure polyethylene specimens increased from 7.7 MPa to 12.1 MPa after gamma radiation. The average bending strength of the specimens containing 30% filler material by weight reached 16.9 MPa from 12.1 MPa based on the gamma radiation. Based on these findings, it could be seen that the physical structure between the matrix and the filler material was directly affected by the gamma radiation, reinforcing the bonding mechanism. As the bonding mechanism is reinforced, the bending strength of the specimens improved from approximately 3.2 to 6.5 MPa.

### 3.4. Polypropylene Bending Strength Findings

**Figure 7:**
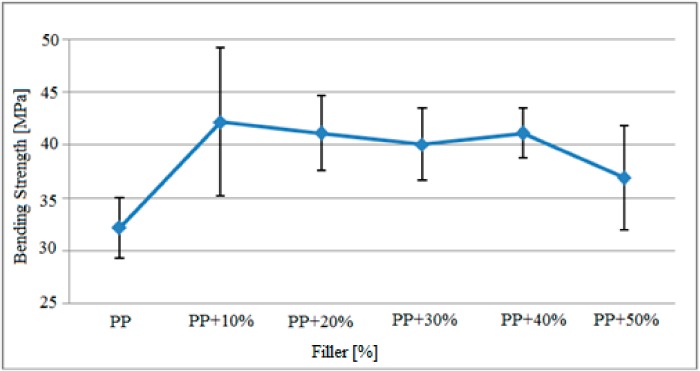
Variation in the bending strength of polypropylene matrix samples after gamma radiation based on the filler material.

Figure 7 depicts the variation in three point bending strength after the filler material reinforced polypropylene was exposed to gamma radiation.

Based on the graph above, the three-point bending strength of the gamma-radiated polypropylene matrix specimens increased in general based on the amount of filler added. While the bending strength values of the pure polypropylene specimens without the filler material were about 32.1 MPa, the bending strength of the specimens containing 10% filler by weight reached the maximum value of 42.1 MPa. The bending strength, which generally varies between 40 and 42 MPa based on the added filler, was measured as 36.9 MPa in specimens with 50% filler. It was observed that the standard deviation range of the specimens was at most 10% for filler material reinforced specimens. It could therefore be argued that specimens containing 10% filler do not mix homogeneously during production. In the other samples, the standard deviation range varied similarly as well.

**Figure 8:**
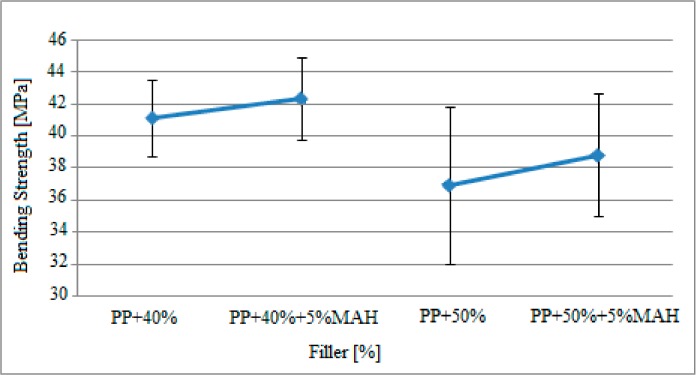
The bending strength findings for filler material reinforced polypropylene containing 5% MAH and exposed to gamma radiation.

The bending strength findings for the “G” and “H” specimens containing 5% MAH (maleic anhydride) and exposed to gamma radiation shown in Table 2 are presented in Figure 8.

In Figure 8, it can be seen that the resistance to vertical loads of the specimens with the same filler ratio exposed to gamma radiation improved due to the maleic anhydride (MAH) content. Based on these findings, the bending strength of specimens containing 40% filler material improved from 41.1 MPa to 42.3 MPa with the effect of 5% MAH. Similarly, the bending strength of the specimens with 50% filler material increased from 36.9 MPa to 38.8 MPa with the effect of 5% MAH.

The findings depicted in Figure 7 and Figure 8 were compared with the results of bending stress results obtained in the study by Kismet (2012) conducted with samples that were not exposed to gamma radiation and are presented in Table 6 for comparison.

Based on the findings mentioned above, it was observed that the resistance of the specimens against vertical loads improved after radiation when compared to the state before radiation. The bending strength of pure polypropylene increased from 31.0 MPa to 32.1 MPa after radiation. Similarly, the bending strength of specimens with 50% filler material increased from 31.3 MPa to 36.9 MPa, increasing by 5.6 MPa based on the gamma radiation. It was also observed that the resistance against vertical loads increased after radiation for the polypropylene matrix specimens containing MAH.

### 3.5. Izod Impact Strength

#### Polyethylene Impact Strength Findings

**Figure 9:**
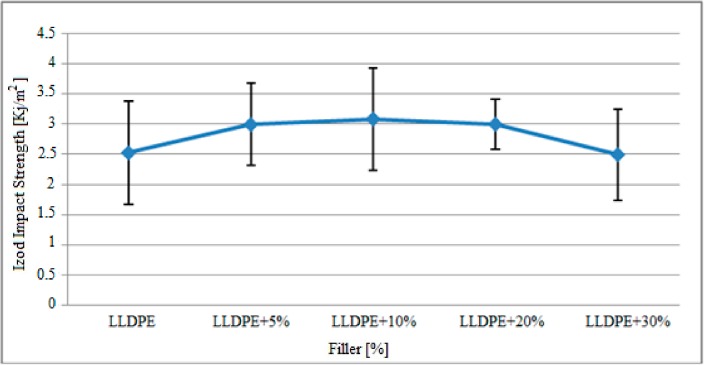
Variation in Izod impact strength of the polyethylene matrix specimens after gamma radiation.

The variation in Izod impact strength of the polyethylene matrix specimens with the mixing ratios shown in Table 1 after gamma radiation is presented in Figure 9.

Based on the graph above, the Izod impact strength of the filler-reinforced polyethylene matrix specimens increased after radiation. With the effect of the gamma radiation, the Izod impact strength of pure polyethylene specimens was approximately 2.52 KJ/m^2^, while the specimens containing 10% and 20% filler material reached the maximum Izod impact strength which was measured at approximately 3.0 KJ/m^2^. However, with the addition of 30% filler, this value decreased to approximately 2.48 KJ/m^2^.

The above mentioned findings given in Figure 9 are presented comparatively with the findings obtained with non-gamma radiated specimens in Table 7.

Based on the findings above, the Izod impact strength of the specimens increased with gamma radiation. In pure polyethylene specimens that were not exposed to gamma radiation, the Izod impact strength was about 2.38 KJ/m^2^, while this value increased to 2.52 KJ/m^2^ after gamma radiation. Similarly, the Izod impact strength of specimens with 30% filler material reinforcement increased from about 2.32 KJ/m^2^ to 2.49 KJ/m^2^ depending on the gamma radiation.

### 3.6. Polypropylene Impact Strength Findings

**Figure 10:**
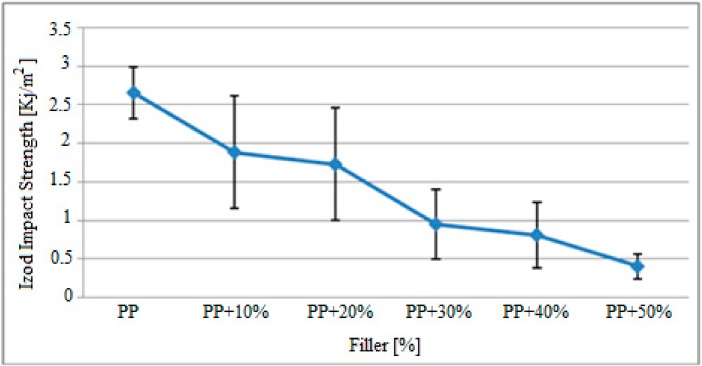
Variation in Izod impact strength of the filler-reinforced polypropylene matrix specimens after gamma radiation.

Variation in Izod impact strength of the filler-reinforced polypropylene matrix specimens after gamma radiation is presented in Figure 10.

Based on the graph above, the Izod impact strengths of the polypropylene matrix specimens exposed to gamma radiation were significantly lower, depending on the filler content. While the impact strength of pure polypropylene samples was approximately 2.65 KJ/m^2^, this value decreased drastically in 50% filler reinforced samples to approximately 0.4 KJ/m^2^. Especially in the samples containing 10% and 20% filler material, the standard deviation range shown in the graph demonstrated that the mixture was not sufficiently homogeneous. These findings are compared with the impact strength values of polypropylene test specimens that were not exposed to gamma radiation in a study by Kismet (2012) and the results are presented in Table 8.

**Figure 11:**
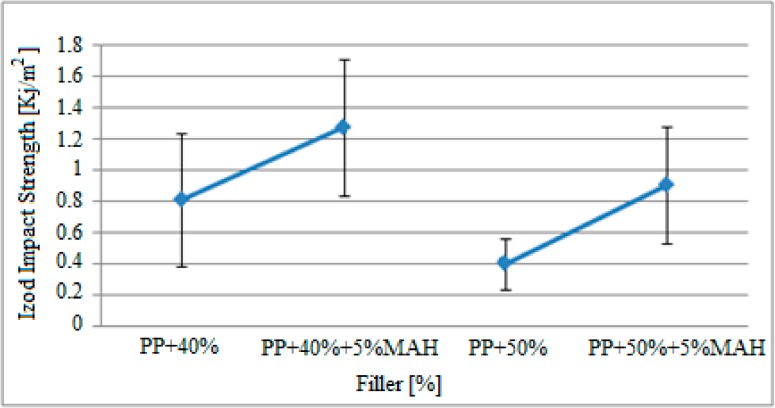
Variation in Izod impact effect findings for 5% MAH-reinforced polypropylene specimens exposed to gamma radiation.

Figure 11 demonstrates the variations in Izod impact strength of specimens with the same filler rate and exposed to gamma radiation as a result of maleic anhydride (MAH) effect. Based on these findings, when 5% MAH is added to the polypropylene matrix reinforced with 40% and 50% filler material, the Izod impact strength of the specimens increased. This increase is approximately between 0.8 KJ/m^2^ and 1.27 KJ/m^2^ in specimens containing 40% filler material. For specimens containing 50% filler material, the Izod impact strength increased from 0.4 KJ/m^2^ to 0.9 KJ/m^2^ by 0.5 KJ/m^2^.

Comparison of the results observed in Figure 10 and Figure 11 and the results for specimens that were not exposed to gamma radiation and presented in Kısmet (2012) are presented in Table 8.

The comparison of pre- and post-radiation findings demonstrated that the Izod impact strength of the specimens improved with gamma radiation exposure. The impact strength of pure polypropylene specimens increased from 2.5 KJ/m^2^ to 2.65 KJ/m^2^, while the strength of the specimens with 50% filler material increased from 0.15 KJ/m^2^ to 0.4 KJ/m^2^. Furthermore, it was observed that the Izod impact strength increased with the gamma radiation effect in samples that contained MAH.

### 3.7. Scanning Electron Microscope Findings

**Figure 12:**
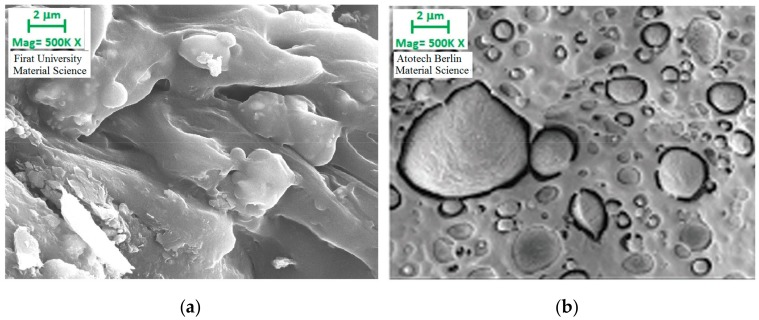
Electron microscope images of 10% filler reinforced polyethylene specimens with (**a**) and without (**b**) gamma radiation.

Figure 12 demonstrates the electron microscope images of polyethylene matrix samples containing 30% filler before and after gamma radiation exposure. It appears that there were gaps between the matrix and the filler before the gamma exposure (b). Thus, it could be argued that the bonding mechanism was weak. However, when the image of the section after radiation is examined, it appears that the hollow structure has been removed. Therefore, it could be argued that the physical structure was reinforced by the direct impact of the radiation on the matrix and filler material.

**Figure 13:**
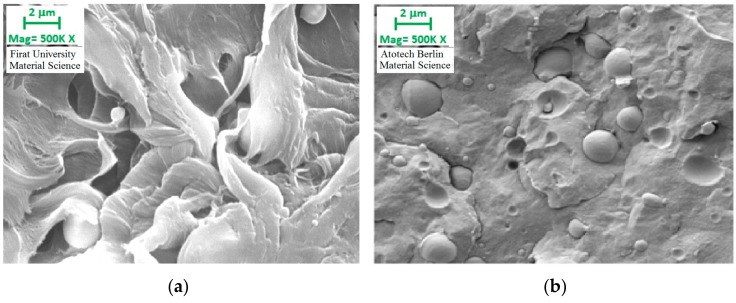
Electron microscope images of 10% filler reinforced polypropylene specimens with (**a**) and without (**b**) (Kısmet, 2012) gamma radiation.

Electron microscope images of 10% filler reinforced polypropylene specimens exposed and not exposed to gamma radiation are presented in Figure 13. Similar to Figure 12 here, the gap between the filler and the matrix material is removed as a result of the gamma radiation and the filler material appeared to form a stronger bonding structure within the polypropylene. Thus, it was found that the physical structure between the matrix and the filler material is directly affected by the gamma radiation, and the bonding mechanism is reinforced as a result.

## 4. Conclusions

In the present study, it was determined that the mechanical properties of electrostatic powder coating reinforced composite material with polyethylene and polypropylene matrix material changed as a result of gamma radiation exposure. These changes were as follows:

It was found that the tensile stress of the electrostatic powder coating reinforced polyethylene and polypropylene specimens that were exposed to gamma radiation decreased with increasing amount of filler material. It could be argued that this was due to the gaps between the matrix and the filler material, observed in the broken sections of the material. The samples were cut from weak sections where these air gaps existed. It was considered that these air gaps occurred during the production (either in the extruder or during the plastic injection). Furthermore, when electron microscope images were examined, it was seen that the matrix and filler material had a weak bonding mechanism and gaps existed between them. This poor bonding mechanism was a factor in reducing the tensile strength of the specimens. However, this structure was somewhat improved with gamma radiation and the tensile strength of the specimens increased by strengthening the bonding mechanism between the filler and the matrix material.

Another mechanical analysis, the bending strength findings demonstrated that the bending strength increased with the increase in filler content in the gamma-radiated specimens, contrary to the tensile stress findings. In the three-point bending test, the force applied to the specimen was perpendicular to both the specimen cross-section and the filler material dispersed in the sample. Thus, the energy generated by the force must either break the filler particles or leave the specimen by passing around the particles. Therefore, as the sample filler content increases, it becomes difficult for the energy to break through the sample and the sample demonstrates a more resilient structure. Again, similar to the tensile strength, the bending strength of samples exposed to gamma radiation demonstrated a significant increase when compared to the bending strength of the specimens that were not exposed to radiation. That is, the bonding mechanism between the matrix and the filler material was reinforced by gamma radiation, and as a result, the strength of the specimens against vertical loads improved.

The Izod impact strength of the polyethylene specimens exposed to gamma radiation increased with the increase in filler content, while it decreased significantly in polypropylene specimens. As a matter of fact, polypropylene is a more brittle material when compared to polyethylene. Furthermore, when electron microscopic images were examined, it was observed that powder coating created a stronger bond structure with polyethylene. However, the Izod impact strength of both polypropylene and polyethylene specimens increased with gamma radiation when compared to the Izod impact strength of the unexposed specimens was they became more resistant to impact. That is to say, as mentioned in the previous two mechanical analyses, the bonding mechanism between the matrix and the filler material was strengthened by the gamma radiation.

Mechanical properties of polypropylene matrix specimens that contained maleic anhydride (MAH) as binding element demonstrated a 10–20% improvement after gamma radiation.

## Figures and Tables

**Table 1:** The amounts and the quantities of filler by weight included in the polyethylene matrix specimens exposed to gamma radiation. LLDPE (low density linear polyethylene).

Standard Tensile Test Samples	Quantity LLDPE (%)	Quantity Filler (%)	Piece
A	100	-	16
B	95	5	15
C	90	10	16
D	80	20	15
E	70	30	16

**Table 2:** The amounts and the quantities of filler by weight included in the polypropylene matrix specimens exposed to gamma radiation. MAH: maleic anhydride; PP: polypropylene.

Standard Tensile Test Samples	Quantity PP (%)	Quantity Filling (%)	Quantity MAH (%)	Piece
A	100	-	-	16
B	90	10	-	1
C	80	20	-	15
D	70	30	-	15
E	60	40	-	15
F	50	50	-	15
G	55	40	5%	15
H	45	50	5%	15

**Table 3:** Comparative presentation of tensile stress results for polyethylene matrix specimens before and after radiation.

Numbers	Tensile Stress (MPa) (Pre-Gamma)	Tensile Stress (MPa) (after Gamma Formation)
LLDPE (Pure)	17.0	18.2
LLDPE + 5% Filler	15.2	16.3
LLDPE + 10% Filler	14.3	15.7
LLDPE + 20% Filler	11.5	13.2
LLDPE + 30% Filler	10.9	11.9

**Table 4:** Comparative presentation of polypropylene matrix specimens tensile stress findings before and after radiation exposure.

Numbers	Tensile Stress (MPa) (Pre-Gamma)	Tensile Stress (MPa) (after Gamma Formation)
PP (Pure)	32.8	34.7
PP + 10% Filler	31.6	32.9
PP + 20% Filler	28.7	29.9
PP + 30% Filler	26.4	27.5
PP + 40% Filler	25.7	26.7
PP + 50% Filler	14.7	17.3
PP + 40% Filling Material + 5% MAH	26.7	27.9
PP + 50% Filling Material + 5% MAH	15.9	19.8

**Table 5:** Comparative presentation of polyethylene matrix specimen bending strength findings before and after radiation.

Numbers	Bending Strength (MPa) (Pre-Gamma)	Bending Strength (MPa) (after Gamma Formation)
LLDPE (Pure)	7.7	12.1
LLDPE + 5% Filler	8.2	13.7
LLDPE + 10% Filler	9.7	16.2
LLDPE + 20% Filler	11.6	15.8
LLDPE + 30% Filler	12.1	16.9

**Table 6:** Comparative presentation of bending strength findings for polypropylene matrix specimens before and after the radiation exposure.

Numbers	Bending Strength (MPA) (Pre-Gamma)	Bending Strength (MPA) (after Gamma Formation)
PP (Pure)	31.0	32.1
PP + 10% Filler	39.0	42.1
PP + 20% Filler	40.5	41.1
PP + 30% Filler	38.1	40.0
PP + 40% Filler	36.1	41.1
PP + 50% Filler	31.3	36.9
PP + 40% Filler + 5% MAH	41.1	42.3
PP + 50% Filler + 5% MAH	36.9	38.8

**Table 7:** Comparative presentation of Izod impact strength findings for polyethylene matrix samples before and after radiation.

Numbers	Izod Impact Resistance (KJ/m^2^) (Pre-Gamma)	Izod Impact Resistance (KJ/m^2^) (after Gamma Formation)
LLDPE (Pure)	2.38	2.52
LLDPE + 5% Filler	2.76	2.90
LLDPE + 10% Filler	2.89	3.08
LLDPE + 20% Filler	2.84	2.99
LLDPE + 30% Filler	2.32	2.49

**Table 8:** Comparative presentation of Izod impact strength findings for polyethylene matrix specimens before and after radiation.

Numbers	Izod Impact Resistance (KJ/m^2^) (Pre-Gamma)	Izod Impact Resistance (KJ/m^2^) (after Gamma Radiation)
PP (Pure)	2.5	2.65
PP + 10% Filler	1.7	1.8
PP + 20% Filler	1.6	1.7
PP + 30% Filler	0.7	0.9
PP + 40% Filler	0.6	0.8
PP + 50% Filler	0.15	0.4
PP + 40% Filler + 5% MAH	0.9	1.27
PP + 50% Filler + 5% MAH	0.5	0.9
